# Nuclear Magnetic Resonance Derived Biomarkers for Evaluating Cardiometabolic Risk in Youth and Young Adults Across the Spectrum of Glucose Tolerance

**DOI:** 10.3389/fendo.2021.665292

**Published:** 2021-05-18

**Authors:** Stephanie T. Chung, Samantha T. Matta, Abby G. Meyers, Celeste K. Cravalho, Alfredo Villalobos-Perez, Joshua M. Dawson, Vandhna R. Sharma, Maureen L. Sampson, James D. Otvos, Sheela N. Magge

**Affiliations:** ^1^ Diabetes, Endocrinology and Obesity Branch, National Institute of Diabetes, Digestive and Kidney Diseases, National Institutes of Health, Bethesda, MD, United States; ^2^ Department of Endocrinology and Diabetes, Children’s National Hospital, Washington, DC, United States; ^3^ National Institutes of Health Clinical Center, National Institutes of Health, Bethesda, MD, United States; ^4^ Laboratory Corporation of America Holdings (LabCorp), Morrisville, NC, United States; ^5^ Division of Pediatric Endocrinology and Diabetes, Johns Hopkins University School of Medicine, Baltimore, MD, United States

**Keywords:** NMR, youth, insulin resistance, cardiometabolic risk, lipoprotein insulin resistance index, GlycA, glucose tolerance

## Abstract

Youth with obesity have an increased risk for cardiometabolic disease, but identifying those at highest risk remains a challenge. Four biomarkers that might serve this purpose are “by products” of clinical NMR LipoProfile^®^ lipid testing: LPIR (Lipoprotein Insulin Resistance Index), GlycA (inflammation marker), BCAA (total branched-chain amino acids), and glycine. All are strongly related to insulin resistance and type 2 diabetes (T2DM) in adults (glycine inversely) and are independent of biological and methodological variations in insulin assays. However, their clinical utility in youth is unclear. We compared fasting levels of these biomarkers in 186 youth (42 lean normal glucose tolerant (NGT), 88 obese NGT, 23 with prediabetes (PreDM), and 33 with T2DM. All four biomarkers were associated with obesity and glycemia in youth. LPIR and GlycA were highest in youth with PreDM and T2DM, whereas glycine was lowest in youth with T2DM. While all four were correlated with HOMA-IR (Homeostatic Model Assessment for Insulin Resistance), LPIR had the strongest correlation (LPIR: r = 0.6; GlycA: r = 0.4, glycine: r = −0.4, BCAA: r = 0.2, all *P* < 0.01). All four markers correlated with HbA1c (LPIR, GlycA, BCAA: r ≥ 0.3 and glycine: r = −0.3, all *P* < 0.001). In multi-variable regression models, LPIR, GlycA, and glycine were independently associated with HOMA-IR (Adjusted R^2^ = 0.473, P < 0.001) and LPIR, glycine, and BCAA were independently associated with HbA1c (Adjusted R^2^ = 0.33, P < 0.001). An LPIR index of >44 was associated with elevated blood pressure, BMI, and dyslipidemia. Plasma NMR-derived markers were related to adverse markers of cardiometabolic risk in youth. LPIR, either alone or in combination with GlycA, should be explored as a non-insulin dependent predictive tool for development of insulin resistance and diabetes in youth.

**Clinical Trial Registration:**

Clinicaltrials.gov, identifier NCT:02960659

## Introduction

Cardiometabolic diseases are a major cause of morbidity and mortality worldwide. Excess adiposity in childhood is an important modifiable risk factor and one of the strongest predictors of future disease in adults (1, 2). Yet global rates of childhood obesity continue to rise unabatedly despite numerous primary prevention campaigns (3). To tackle this growing public health problem, prevention and intervention strategies should be coupled and targeted to youth and young adults at highest risk (4). Current approaches in pediatrics rely heavily on clinical and single laboratory parameters, such as BMI percentile and screening tests for hyperglycemia, to help clinicians diagnose and assess risk for cardiometabolic diseases. Although these tools are useful for the diagnosis of obesity and diabetes among growing children, they are relatively insensitive to risk stratification of insulin resistance, a primary pathophysiologic factor in cardiometabolic diseases (5).

Quantitatively assessing insulin resistance in the clinical arena has been challenging as it requires a test that is simultaneously effective and suitable for widespread clinical use. Although multiple invasive provocative methods are available to measure insulin resistance, none are suitable for routine clinical application. Alternatively, simple fasting blood tests to measure glucose and insulin are more economical and less labor intensive. For example, the homeostasis model of insulin resistance (HOMA-IR) is frequently used in research and epidemiological analyses for metabolic risk stratification (6, 7). However, outside of the research arena, its clinical application is limited because of lack of standardization of insulin assays resulting in poor reproducibility across clinical laboratory platforms (8, 9).

Four potential nuclear magnetic resonance (NMR)-derived markers—lipoprotein insulin resistance index (LPIR), GlycA (a composite marker of inflammation), total branched chain amino acids (BCAA), and glycine—have emerged as useful tools in adults with the prospect that they could augment existing clinical phenotypes to improve cardiometabolic risk stratification (10, 11). These surrogate markers of insulin resistance, derived from NMR analyzers, are attractive for routine clinical assessments because they provide reliable quantification without the need for specialized assays or time-consuming resources. These biomarkers are efficiently and inexpensively obtained from automated NMR analyzers that are already routinely used for clinical lipid and lipoprotein analysis (12).

Notably, despite the potential for improving care, there is a paucity of data on the use of these NMR biomarkers across the spectrum of insulin resistance and glucose tolerance in the pediatric population, especially in minority youth at risk for T2DM. NMR biomarkers of insulin resistance are important to explore in youth because they would facilitate estimation of an insulin independent marker of insulin resistance. Dyslipidemia of insulin resistance is a key risk factor for cardiovascular disease, precedes the onset of dysglycemia, and reflects adipose tissue and hepatic responses to insulin signaling defects (13). Since adolescence is associated with physiologic changes in lipid profiles, it is important to investigate the association of these markers with glycemia and insulin resistance in youth. We hypothesized that LPIR, GlycA, and total BCAA would be higher in youth with abnormal glucose tolerance and would positively correlate, while glycine would be negatively correlated with insulin resistance. Our primary objectives were to compare these four NMR biomarkers (LPIR, GlycA, BCAA, and glycine) in youth with and without obesity across the spectrum of glucose tolerance and determine the association of these four biomarkers with insulin resistance and glycemia.

## Materials and Methods

This was a secondary analysis of participants enrolled in two observational cohort cross-sectional studies that were designed to evaluate the pathophysiology of T2DM in youth: The MIGHTY study (Metformin Influences Gut Hormones in Youth) cohort was recruited from two clinical sites (Baylor College of Medicine, Houston TX and National Institutes of Health (NIH), Bethesda, MD) and The Dyslipidemia and Cardiovascular (CV) Risk Factors in Pediatric Obesity and Type 2 Diabetes study cohort was recruited from the Children’s Hospital of Philadelphia (CHOP). Data on primary analyses for the cohorts have been previously published (14, 15). The studies were approved by the Institutional Review Boards at Baylor College of Medicine, NIH, and CHOP. All enrollees 18 years or older and parents of participants <18 years gave written informed consent prior to participation. Participants <18 years gave verbal consent and signed a written assent.

**Figure 1:**
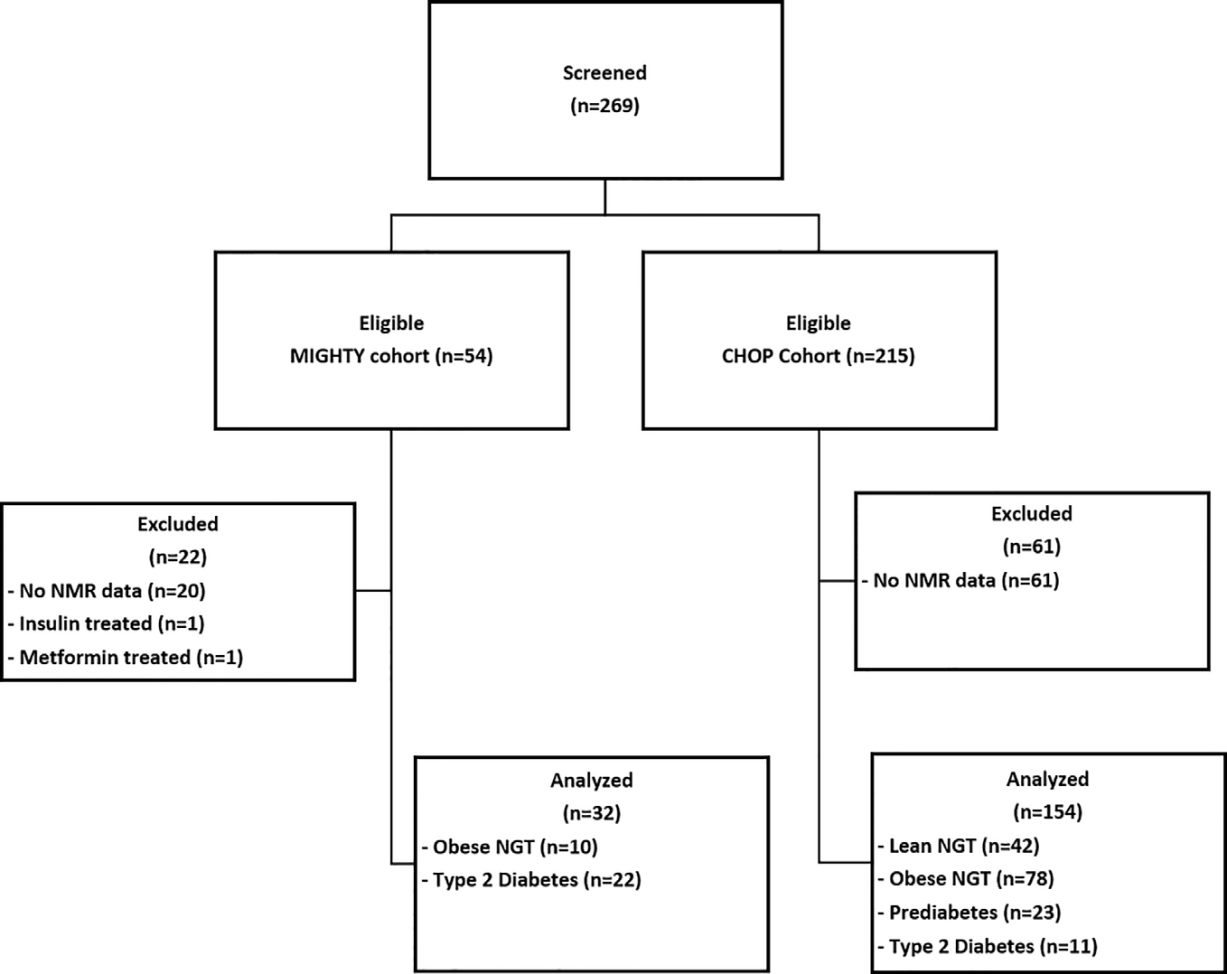
Participant Flow Diagram. MIGHTY, Metformin Influences Gut Hormone Study in Type 2 Diabetes Youth; CHOP, Children’s Hospital of Philadelphia; NMR, nuclear magnetic resonance; NGT, normal glucose tolerant; PreDM, prediabetes; T2DM, type 2 diabetes.

All youth who had a baseline evaluation for fasting lipoprotein profile were included in this analysis (**Figure 1**). Youth were pubertal (Tanner 2–5), 78% African American (self-reported), age 10–25 years, had no known major chronic illnesses (except T2DM and/or obesity), were not pregnant, and were not on medications known to affect insulin sensitivity (*e.g.* statins, vitamin A, or oral steroids). Tanner stage was determined based on breast examination in girls and testicular examination in boys. The following conversion of testicular volume to Tanner stage was used: <4 cc: Stage 1; 4–6 cc: Stage 2; 7–10 cc: Stage 3; 11–15 cc: Stage 4; >15 cc: Stage 5. Pubic hair and bone age were not used to characterize pubertal stage. Blood pressure was measured in the seated patient’s right arm after 10–15 minutes at rest. Blood pressure percentiles for age, sex, and height were calculated based on American Academy of Pediatrics 2017 guidelines (16, 17). BMI was calculated as weight in kilograms divided by height in meters squared, and BMI percentiles were assessed using age and sex specific BMI reference data (18). Height measurements were repeated three times, and the average value was utilized for BMI calculations. Obesity was defined as BMI ≥95%ile and lean as BMI: 5–85%ile for age and using CDC growth charts. Youth with T2DM and prediabetes (PreDM) were previously diagnosed by their physician or by a 2-h standard oral glucose tolerance test (OGTT) and American Diabetes Association criteria; the same criteria were used to classify youth as having NGT (19). Briefly, the ADA defines prediabetes as fasting plasma glucose levels of 100 mg/dl to 125 mg/dl or 2-h plasma glucose during a 75-g oral glucose tolerance test of 140 mg/dl to 199 mg/dl or an HbA1c of 5.7–6.4%. Diabetes is defined as a fasting glucose of ≥126 mg/dl or a 2-h plasma glucose ≥200 mg/dl during OGTT or an HbA1c ≥6.5%, or in patients with classic symptoms of hyperglycemia or hyperglycemic crisis, a random plasma glucose ≥200 mg/dl.

**Figure 1** details the patient flow diagram: 186 youth were eligible to participate in this secondary analysis: 42 lean control, 88 obese NGT, 23 obese PreDM, 33 obese T2DM. In the youth with T2DM, nine were drug-naïve and 24 were treated with metformin which was discontinued 4–7 days prior to the assessment. Six participants from the CHOP cohort were regularly treated with insulin but were withdrawn from their insulin treatment prior to fasting blood draw as follows: 36 h for insulin glargine, 24 h for non-protamine Hagedorn (NPH) insulin, and 12 h for short-acting insulin prior to baseline evaluation. All participants had blood samples for plasma glucose, insulin, lipid panel, and lipoprotein profile obtained after a 10–12 h overnight fast.

### Biochemical Analyses and Calculations

#### Lipid and Lipoprotein Analyses

All plasma samples were obtained at the baseline visit after participants fasted overnight. Samples were processed and stored at −80°C prior to analysis. Lipoprotein particle size and subclass concentrations were measured by the amplitudes of the lipid-methyl group NMR signals and reported in particle concentration units (nmol/L). Analysis was conducted with a 400 Mhz proton NMR Profiler and Vantera Clinical Analyzer platforms (20, 21). The lipid methyl signal envelope algorithm analysis (LP4.17) was used to characterize particle size and concentration. Because of the combination of two different study cohorts using different lipid measurement methodologies, we did not feel it scientifically sound to combine the original lipid values. Therefore, for this analysis we estimated the lipid values from partial least square regression models using a validated methodology (12).

The lipid panel (total cholesterol, high-density lipoprotein cholesterol, and triglyceride concentrations), apolipoprotein B, and apolipoprotein A concentrations were estimated from partial least square regression models of lipid methyl and methylene region (0.494–1.592 ppm; 1,600 data points) of serum NMR LipoProfile spectra (12). Estimated low-density lipoprotein cholesterol values were calculated as follows (22):

***LDL-C (mg/dl) = (TC/0.948) − (HDL-C/0.971) − (TG/8:56 + TG × Non-HDL-C/2140 − TG^2^/16,100 − 9:44).***

The lipoprotein insulin resistance index (LPIR) was calculated from a composite score of six NMR lipoprotein (LP) parameters scored 0–100, with higher score indicating higher insulin resistance (23–25). BCAA, glycine, and GlycA were quantified from spectral deconvolution of signal amplitudes using *NMR LipoProfile^®^.* The three BCAAs (valine, leucine, isoleucine) were quantified from NMR signals. Since levels of valine, leucine, and isoleucine are highly correlated, their sum (“BCAA”) is reported for ease of epidemiologic investigation. Thus, BCAA concentrations were calculated as the sum of valine, leucine, and isoleucine concentrations in each participant.

#### Insulin and Metabolites

Serum insulin concentrations were measured with standardized assays at each institution (reported coefficient variations ≤10%); NIH: Roche Cobas 6000 analyzer (Roche Diagnostics, Indianapolis, IN), CHOP: ELISA, ALPCO Diagnostics (Salem, NH), and Baylor: Elecsys 1010 Analyzer (Roche Diagnostics, Indianapolis, IN). HbA1c was measured with high-performance liquid chromatography. Data for HbA1c (n = 7), glucose (n = 1), and insulin (n = 2) in some participants were not available due to technical difficulties.

#### Calculations

The equation used to calculate HOMA-IR was

$$\frac{\text{fasting}\;\text{blood}\;\text{glucose}\;(\text{mg}/\text{dl})*\text{fasting}\;\text{insulin}\;(\text{mIU}/\text{L})}{405}$$

HOMA-IR strongly correlates with insulin resistance measured by the gold-standard hyperinsulinemic clamp and has been evaluated in individuals with and without diabetes (r > 0.6, *P* < 0.001) (26, 27). The advantage of HOMA-IR in this research protocol was the ability to evaluate a large number of youths without invasive blood sampling required for clamp measurements and rigor and reliability of insulin measurements using standardized research insulin assays. To account for calibrations to the contemporary insulin assays and the insulin-glucose dynamics with hyperglycemia, we also used a corrected nonlinear model (the HOMA2 Calculator from ^©^The University of Oxford 2004–2021) to estimate HOMA-2 (28).

### Statistical Analyses

Data are presented as mean ± SD, except where otherwise indicated. Insulin, triglycerides, BCAA, and HOMA-IR were natural log transformed prior to analysis. Continuous variables were compared across groups with one-way analysis of variance with Bonferroni corrections and categorical variables using Chi-squared tests. Spearman correlations (r) were used to determine the association of biomarkers with HbA1c and HOMA-IR. Multi-linear regression models were created to determine the relationship between HOMA-IR or HbA1c (outcome variables) and biomarkers (predictor variables), adjusted for Tanner stage, age, race/ethnicity, and sex. These covariates were chosen because they are known mediators of insulin resistance. To test for multi-collinearity among independent variables in the regression models we used variance inflation factors. The variance inflation factors were <2 indicating weak correlation among variables. *P*-value <0.05 was considered statistically significant. All statistical analyses were performed using STATA (version 16.1; Stata Corp, College Station, TX).

## Results

### Participant Demographic and Metabolic Characteristics

Youth were 14.7 ± 1.5years, 49.5% female, 89.7% Tanner 4–5, and 78.0% African American (**Table 1**). By design, BMI was higher and comparable between obese NGT, obese PreDM, and obese T2DM groups *vs.* lean NGT. There was no significant age difference among the four groups. Youth with T2DM had the highest HbA1c, fasting glucose, and fasting insulin. HOMA-IR and HOMA2-IR were similar in obese PreDM and obese T2DM and were higher compared to obese NGT and lean NGT. Similarly, the dyslipidemic pattern (elevated triglycerides, higher apolipoprotein B, higher LDL cholesterol, and lower HDL) was similar in obese PreDM and T2DM and higher compared to lean and obese NGT.

**Table 1:** Participant Demographic and Metabolic Characteristics.

Variable	Overall (n = 186)	Lean NGT (n = 42)	Obese NGT (n = 88)	Obese-PreDM (n = 23)	Obese T2DM (n = 33)	Overall p-value
**Demographic**						
**Age (years)**	14.65 ± 1.53	14.7 ± 1.31	14.5 ± 1.46	14.4 ± 1.41	15.0 ± 1.98	0.34
**Female**	92 (49.5)	21 (50)	49 (55.7)	9 (39.1)	13 (39.4)	0.30
**Race/Ethnicity**
**African American**	145 (78)	34 (81.0)	65 (73.9)	21 (91.3)	25 (75.8)	
**White**	14 (7.5)	2 (4.76)	8 (9.1)	1 (4.4)	3 (9.1)	0.13
**Hispanic/Latino**	10 (5.4)	0 (0.00)	6 (6.8)	0 (0.00)	4 (12.1)	
**Mixed**	14 (7.5)	6 (14.3)	6 (6.8)	1 (4.4)	1 (3.0)	
**Unknown**	3 (1.6)	0 (0.00)	3 (3.4)	0 (0.00)	0 (0.00)	
**Tanner Stage**						
**2 and 3**	19 (10.3)	2 (4.8)	10 (11.4)	6 (27.3)	1 (3.0)	
**4 and 5**	166 (89.7)	50 (95.2)	78 (88.6)	16 (72.7)	32 (97.0)	**0.02**
**BMI (kg/m^2^)**	32.2 ± 8.8	20.0 ± 1.85^a,b^	34.9 ± 5.77 ^a^	36.1 ± 7.4 ^b^	38.2 ± 7.14 ^a^	**<0.0001**
**Systolic BP (mmHg)**	113.7 ± 12.0	109.5 ± 12.2^a^	112.0 ± 10.0^b^	115.8 ± 13.8	122.2 ± 11.7 a^,b^	**<0.0001**
**Diastolic BP (mmHg)**	63.2 ± 7.6	64.0 ± 8.06	61.6 ± 6.37	61.7 ± 6.04	67.6 ± 9.59	**0.0010**
**Systolic BP >90^th^ percentile**	32 (17)	4 (10)	10 (11)	5 (22)	13 (40)	**0.002**
**Diastolic BP >90^th^ percentile**	5 (3)	1 (2)	0 (0)	0 (0)	4 (14)	**0.001**
**Metabolic (Fasting)**						
**Glucose (mg/dl)**	95.4 ± 22.1	86.0 ± 5.8^a^	88.4 ± 5.82^b^	96.3 ± 6.89^c^	124.9 ± 38.5 a^,b,c^	**<0.0001**
**Insulin (μU/L)**	22 ± 19.8	8.28 ± 4.13^a,b^	21.3 ± 12.5^a^	29.7 ± 15.4 ^b^	39.9 ± 32.1 ^a^	**<0.0001**
**HOMA-IR**	3.8 (2.1, 6.8)	1.7 (1.1, 2.0)^a,b^	4.0 (2.8, 5.8) ^a,b^	7.5 (3.5, 9.7)^a^	10.2 (4.7, 16.7) ^b^	**<0.0001**
**HOMA2-IR**	1.9 (1.1, 3.3)	0.91 (0.58, 1)^a,b^	2.0 (1.5, 2.8) ^a,c^	3.5 (1.5, 4.9) ^b^	4.1 (1.9, 5.7) ^a,c^	**<0.0001**
**Hemoglobin A1c (%)**	5.60 ± 0.74	5.27 ± 0.30 a	5.35 ± 0.30^b^	5.47 ± 0.43 ^c^	6.77 ± 0.90 a^,b,c^	**<0.0001**
**Total Cholesterol (mg/dl)**	135 ± 26	131 ± 23	134 ± 25	135 ± 28	143 ± 31	0.223
**Triglycerides (mg/dL)**	97 (75, 133)	85 (67, 100)^a,b^	95.5 (71, 130)^c^	121 (86, 155)^a^	122 (95, 179) ^b,c^	**0.0001**
**HDL Cholesterol (mg/dl)**	47 ± 11	58 ± 10^a,b^	46 ± 10^a^	42 ± 7^b^	41 ± 8^a^	**<0.0001**
**LDL Cholesterol (mg/dl)**	64 ± 20	54 ± 17^a,b^	65 ± 17^a^	66 ± 22	73 ± 24^b^	**0.0003**
**Apolipoprotein A1 (mg/dl)**	112.73 ± 17.2	128.3 ± 15.7^a,b,c^	110.2 ± 14.7^a^	107.0 ± 12.3^b^	103.6 ± 15.9^c^	**<0.0001**
**Apolipoprotein B (mg/dl)**	57 ± 17	46 ± 12^a,b^	57 ± 14^a,c^	61 ± 19^b^	67 ± 19^a,c^	**<0.0001**

Data are n (%), mean ± SD or median (25th, 75th percentiles) Groups with the same letter are significantly different (Bonferroni post hoc analysis, p<0.05). BMI, body mass index; BP, blood pressure; HDL, high-density lipoprotein; LDL, low-density lipoprotein; Ln, natural logarithm; HOMA-IR, Homeostatic Model Assessment for Insulin Resistance; HOMA2-IR, revised homeostatic model of insulin resistance. Continuous variables compared with one-way analysis of variance and Bonferroni correction. Insulin, HOMA-IR, HOMA2-IR, and triglycerides natural log-transformed prior to analysis. Categorical variables compared with Chi-squared tests.

Bolded values indicate significant p-values < 0.05.

### NMR Biomarkers

**Figure 2:**
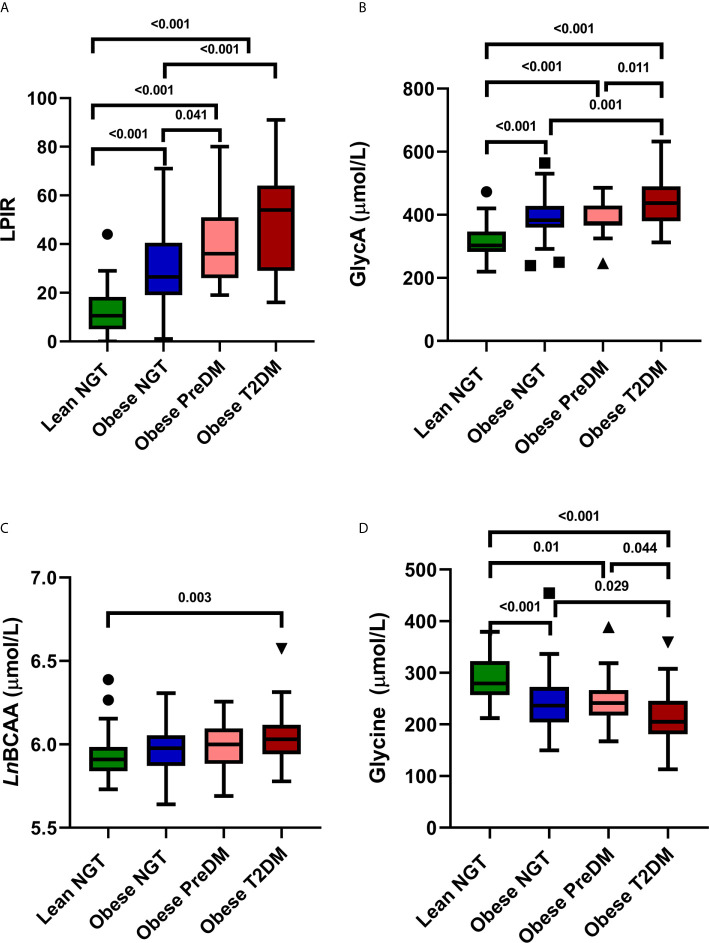
NMR Biomarkers in Youth. Tukey Box and Whisker plots of LPIR score **(A)** GlycA **(B)** Total *Ln*BCAA **(C)** and Glycine concentrations **(D)** in lean NGT (green), obese NGT (blue), obese PreDM (pink) and obese T2DM (red). Groups were compared with one-way ANOVA with Bonferroni corrections. LPIR, lipoprotein insulin resistance score; *Ln*BCAA, natural log transformed branched chain amino acids; NGT, normal glucose tolerance; PreDM, prediabetes; T2DM, type 2 diabetes.

The median LPIR was 26 (range 0–91) and was higher in youth with obesity compared to lean (**Figure 2A**). Among youth with obesity, there was also variation in LPIR scores: LPIR was lower in obese NGT compared to obese PreDM and T2DM (*P* < 0.01), but there was no difference in LPIR between obese PreDM and T2DM (*P* = 0.21). Median GlycA was 376 μmol/L (range 239–574) and highest in obese T2DM compared to the other three groups (**Figure 2B**). Total BCAA was comparable across groups (median 392 μmol/L, range 296–595), although total BCAA was higher in obese T2DM compared to lean NGT, *P* = 0.003 (**Figure 2C**). As expected, glycine was inversely related to glucose tolerance status in a stepwise fashion (median 240 μmol/L, range 123–388, **Figure 2D**). The biomarkers did not differ by age (**Supplemental Table 1**), African ancestry (**Supplemental Table 2**), sex, or Tanner stage (data not shown).

LPIR, GlycA, and total BCAA were positively correlated, and glycine was negatively correlated with HOMA-IR (LPIR: r = 0.6; GlycA: r = 0.4, glycine: r = −0.4, BCAA: r = 0.2, all *P* < 0.01). All four markers correlated with HbA1c (LPIR, GlycA, BCAA: r ≥ 0.3 and glycine: r = −0.3, all *P* < 0.001) (**Supplemental Table 3**). Among the four biomarkers, LPIR had the strongest correlation with both HbA1c and HOMA-IR/HOMA2-IR (r = 0.48 and r ≥ 0.60, respectively). In a combined model using all four biomarkers, LPIR, GlycA, and glycine were independently associated with HOMA-IR (Adjusted R^2^ = 0.473, P < 0.001, **Table 2**). In a separate model, LPIR, glycine, and BCAA were independently associated with HbA1c (Adjusted R^2^ = 0.33, P < 0.001, **Table 2**). To explore whether LPIR could discriminate individuals with highest risk, we divided LPIR into quartiles and compared patient metabolic and demographic variables (**Table 3**). Participants in quartile 4 with an LPIR score of >44 had significantly higher BMI, blood pressure, and metabolic markers of dyslipidemia and dysglycemia (*P* < 0.001).

**Table 2:** Association of NMR-derived biomarkers with HOMA-IR and HbA1c.

	*Ln* HOMA-IR (n = 181)****			HbA1c (n = 176)****		
	Adjusted R^2^ = 0.4733, P < 0.001			Adjusted R^2^ = 0.293, P < 0.001		
	β	SE	95% CI	β	SE	95% CI
**LPIR**	0.202	0.003	0.014, 0.026	0.012	0.003	0.007, 0.018
GlycA	0.002	0.001	0.0008, 0.004	0.001	0.001	−0.001, 0.002
***Ln* BCAA**	0.537	0.34	−0.133, 1.207	1.016	0.355	0.316, 1.717
Glycine	−0.002	0.001	−0.004, −0.0006	−0.003	0.001	−0.005, −0.001

Multi-linear regression models were adjusted for age, Tanner stage, self-reported race/ethnicity, and sex. NMR, nuclear magnetic resonance; HOMA-IR, homeostatic model of insulin resistance; HbA1c, hemoglobin A1c; Ln, natural logarithm.

**Table 3:** Participant demographic and metabolic characteristics and Lipoprotein Insulin Resistance Index Score.

Variable	1^st^ Quartile	2^nd^ Quartile	3^rd^ Quartile	4^th^ Quartile	P-value
	0–16	17–26	28–44	>44	
	n = 47	n = 49	n = 44	n = 46	
Demographic					
**Age (years)**	14.5 ± 1.5	15 ± 1.4	14.4 ± 1.2	14.7 ± 1.9	0.2881
**Female**	27 (57.5)	27 (55.1)	18 (40.9)	20 (43.5)	0.288
**Race**	40 (85.1)	38 (77.6)	35 (79.6)	32 (69.6)	**0.018**
**African American White**	1 (2.13)	8 (16.3)	3 (6.8)	2 (4.35)	
**Hispanic/Latino**	2 (4.26)	0 (0.00)	1 (2.27)	7 (15.2)	
**Mixed**	4 (8.51)	3 (6.12)	3 (6.82)	4 (8.7)	
**Unknown**	0 (0.00)	0 (0.00)	2 (4.55)	1 (2.17)	
**Tanner Stage**					
**2 and 3**	1 (2.13)	3 (6.25)	8 (18.2)	7 (15.2)	
**4 and 5**	46 (97.9)	45 (93.8)	36 (81.8)	9 (84.8)	**0.038**
**Prediabetes**	0 (0)	6 (12.2)	10 (22.7)	7 (15.2)	**<0.001**
**Type 2 diabetes**	2 (4.3)	5 (10.2)	5 (11.4)	21 (45.7)	**<0.001**
**BMI (kg/m^2^)**	24.5 ± 7.18	31.8 ± 7.19	35.0 ± 7.70	38.0 ± 7.04	**<0.001**
**Systolic BP (mmHg)**	111.0 ± 12.7	111.0 ± 10.7	115.9 ± 12.0	117.2 ± 11.8	**0.0165**
**Diastolic BP (mmHg)**	64.1 ± 8.8	61.8 ± 6.5	62.4 ± 6.6	65 ± 8.7	0.15
**Systolic BP > 90^th^ percentile**	6 (12.8)	5 (10.4)	10 (22.7)	11 (24)	0.205
**Diastolic BP > 90^th^ percentile**	1 (2)	0 (0)	0 (0)	4 (9)	**0.024**
Metabolic (Fasting)					
**Glucose (mg/dl)**	88.5 ± 9.8	90.2 ± 9.1	92.9 ± 12.4	110.2 ± 36.7	**<0.00001**
**Insulin (μU/L)**	9.2 (5.9, 15.1)	14.5 (10.2, 17.5)	23.6 (12.3, 32.3)	35.5 (19.6, 46.7)	**<0.00001**
**HOMA-IR**	2.0 (1.1, 3.2)	3.5 (2.1, 4.0)	5.4 (2.9, 7.3)	8.2 (4.6, 15.2)	**<0.0001**
**HOMA2-IR**	1.0 (0.6, 1.7)	1.6 (1.1, 1.9)	2.7 (1.4, 3.5)	3.9 (2.4, 5.4)	**<0.0001**
**Hemoglobin A1c (%)**	5.32 ± 0.32	5.45 ± 0.50	5.47 ± 0.53	6.18 ± 1.05	**<0.001**
**Total Cholesterol (mg/dl)**	145.4 ± 23.7	122.3 ± 23.8	127.5 ± 21.4	145.2 ± 27.3	**<0.001**
**Triglycerides (mg/dl)**	88 (70, 109)	80 (60, 93)	104 (84, 130)	159 (122, 196)	**<0.001**
**HDL Cholesterol (mg/dl)**	60.1 ± 9.70	45.8 ± 7.70	42.9 ± 6.74	39.1 ± 5.9	**<0.001**
**LDL Cholesterol (mg/dl)**	64.9 ± 19.5	58.1 ± 19.7	59.4 ± 16.9	72.5 ± 20.1	**0.001**
**Apolipoprotein A (mg/dl)**	129.4 ± 15.2	108.5 ± 14.6	107.8 ± 13.1	104.9 ± 13.9	**<0.0001**
**Apolipoprotein B (mg/dl)**	53.4 ± 13.9	50.3 ± 15.0	54.5 ± 14.3	68.6 ± 17.2	**<0.001**

Data are n (%), mean ± SD or median (25th, 75th percentiles) BMI, body mass index; BP, blood pressure; HDL, high-density lipoprotein; LDL, low-density lipoprotein; HOMA-IR, Homeostatic Model Assessment for Insulin Resistance; HOMA2-IR, revised-model Homeostatic Model of Insulin Resistance. Continuous variables compared with one-way analysis of variance and Bonferroni correction. Insulin, HOMA-IR, HOMA2-IR, and triglycerides natural log-transformed prior to analysis. Categorical variables compared with Chi-squared tests.

Bolded values indicate significant p-values <0.05.

## Discussion

This analysis confirms the association of NMR biomarkers (LPIR, GlycA, BCAA, and glycine) with insulin resistance and glycemia and provides new information on the distribution of these variables in a predominantly African American youth and young adult cohort who were at risk for or who had T2DM. Although each of the four biomarkers was associated with insulin resistance and HbA1c, the strengths of the association differed by BMI and glucose tolerance category. Youth with preDM and T2DM had the highest levels of LPIR and GlycA and the lowest values for glycine—a biochemical profile consistent with high cardiovascular risk in adults. Moreover, LPIR had the strongest and most robust associations with insulin resistance and glycemia (HbA1c) compared to the other three biomarkers. In contrast, BCAA was only weakly correlated with glycemia and insulin resistance. Although higher total BCAA concentrations were observed in youth with obesity, BCAA levels did not differ by glucose tolerance category. Therefore, of the four biomarkers evaluated, LPIR and GlycA would be ideal candidates for long term evaluation as predictor(s) of cardiometabolic diseases in youth and young adults.

Identifying biomarkers of insulin resistance that can be universally applied across race/ethnic populations, regardless of age, is of high clinical significance and urgently needed to simplify and refine primary prevention of cardiovascular diseases (29). Current invasive methodologies for measuring insulin resistance reliably assess hepatic and peripheral tissue responsiveness to insulin, but the extensive time and resources required to perform these metabolic assessments relegate these techniques to the research arena (8). In contrast, each of the four biomarkers evaluated in this study could be efficiently and inexpensively obtained from automated NMR analyzers that are already routinely used for clinical lipid and lipoprotein analysis (12). They are indirect measures of insulin resistance and were chosen because of their potential to identify the tissue-specific effects of insulin resistance, independent of the biological and assay variations in insulin concentrations.

LPIR is a non-insulin dependent fasting test that quantifies one of the earliest manifestations of insulin resistance—dyslipidemia characterized by elevations in triglyceride and lower high-density lipoprotein cholesterol concentrations. LPIR is already an emerging diagnostic and risk stratification tool in adults (30, 31) because elevations in LPIR precede the development of abnormal glucose tolerance (23, 32). However, its diagnostic and predictive ability in youth is unknown. In the current study, we show that LPIR progressively increased across glucose tolerance categories and was associated with insulin resistance and glycemia. Furthermore, an LPIR score of >44 identified individuals with the highest biochemical and demographic markers of cardiometabolic risk. A previous analysis in youth demonstrated an association of LPIR with insulin resistance in a healthy population before and after an exercise intervention (33). The current study confirms the positive association of LPIR with insulin resistance and extends those findings to youth at highest risk (youth with preDM and T2DM). Prior data also suggest that LPIR may be more sensitive to underlying lipid changes associated with adiposity; Magge et al., 2019 found that in youth with Down syndrome compared to controls, LPIR was higher despite no differences in fasting insulin concentrations or HOMA-IR, even when adjusted for demographics, pubertal stage and BMI z-score (34).

Similar to our findings in youth, an LPIR of >44 was associated with the highest cardiometabolic risk in adults and could be used to predict 20 year progression to T2DM (24, 31). The advantage of using LPIR resides with its simplicity in use and its potential as a risk stratification and therapeutic monitoring tool. Due to the cross-sectional nature of this analysis, we were unable to evaluate the predictive ability of LPIR for future cardiometabolic disease in youth. However, taken together, our findings and others, support using a universal cut-point of ≥45 to denote increased cardiometabolic risk in children and adults.

A notable strength of our analyses was identifying the strong association of LPIR with obesity and glycemia in youth of African ancestry. Although simple lipid indices, such as the TG:HDL ratio, have been proposed as clinical biomarkers of insulin resistance in pediatric and adult cohorts (35), these ratios are weak correlates of insulin resistance, especially in African ancestry individuals, who are known to have lower triglyceride concentrations but greater insulin resistance (25, 36). LPIR strongly correlates with the gold-standard hyperinsulinemic euglycemic clamp across a range of ethnicities (23).

An important outstanding question is whether LPIR could be superior to HOMA-IR in risk prediction. HOMA-IR has been used across pediatric populations, but its ability to risk discriminate and predict intervention response in individuals with prediabetes with declining beta-cell function is controversial (26, 29, 37, 38). This study determined the relationship of NMR biomarkers with this existing research and epidemiological tool but was not designed to compare the diagnostic or predictive accuracy of LPIR *vs.* HOMA-IR for future cardiometabolic risk prediction. Prospective analyses are needed to extensively evaluate the predictive potential, sensitivity, and specificity of LPIR cutoffs in children and young adults.

Next, we demonstrated that GlycA correlated with insulin resistance, and there was a stepwise increase in GlycA across categories of obesity and glucose tolerance (**Figure 2B**). Some, but not all studies (39), have identified GlycA as an independent correlate of HOMA-IR, with the added advantage that GlycA had low intra-individual variability in youth and adults compared to other inflammatory markers such as C-reactive protein (33, 40, 41). GlycA is a promising assay because it is an integrated marker of inflammation derived from a composite signal of N-acetyl methyl group resonances from several of the most abundant inflammation response serum proteins (11). In population-based studies, elevated levels were indicators of chronic asymptomatic systemic inflammation from childhood to adulthood (42). However, its value in predicting cardiometabolic diseases in youth is unclear, and in prior analysis, it was not related to exercise-induced improvements in insulin resistance in a cohort of Latino adolescents (39). In agreement with adult studies, our study findings demonstrate a moderately strong correlation of GlycA with insulin resistance and provide new knowledge about the relationship of this inflammatory marker in youth with abnormal glucose tolerance.

Total BCAA is another serum biomarker that has been extensively studied in the last few decades. In this study, we examined the NMR derived total BCAA and showed that BCAA was a weak correlate of insulin resistance across categories of glucose tolerance in our cohort. Our findings agree with previous studies in adults; total BCAA concentrations are elevated in T2DM, but because of its relatively weak correlation with insulin resistance and glycemia, it is not a readily used clinical risk tool (43, 44). Data linking BCAA with insulin resistance is conflicting, especially in cross-sectional studies in which dietary intake is not controlled (43, 45). High BCAA concentrations in individuals with obesity and insulin resistance may be caused by abnormal catabolism. Reduced catabolism, related to impaired branched-chain *α*-keto-acid dehydrogenase, is linked to insulin resistance in murine models (46). Alternatively, BCAAs are essential amino acids whose concentrations directly correlate with dietary intake, and plasma levels may also be altered by differences in microbiome composition and anti-diabetic medications such as metformin therapy (45, 47, 48). Given the multiple lifestyle and environmental factors that influence BCAA concentrations, our data posit that NMR-derived BCAA concentrations are not robust markers of insulin resistance or worsening glycemia in youth. The weak relationship of BCAA with insulin resistance was observed in our cohort despite strict protocols to minimize any effects of medication.

Lastly, we provide novel information on glycine concentrations in youth. Glycine is a relatively new biomarker that is a non-essential amino acid associated with reduced incidence of coronary heart disease and lower risk of T2DM (49, 50). This is the first study, to our knowledge, that has systematically evaluated the relationship of glycine and demonstrated a weak inverse relationship with insulin resistance and glycemia in youth. Despite a small range, glycine was significantly different between each of the four groups and lowest in youth with T2DM. However, the effect size of the differences between groups was small, and the underlying mechanisms of this inverse relationship are not clear and need further validation. Murine data suggest that glycine is important for waste elimination of excess free fatty acid and BCAA catabolism (51, 52). Alternatively, glycine supplementation has been associated with improved insulin secretion and inhibition of inflammation (50). Therefore, glycine’s role as a biomarker and indicator of insulin resistance, especially in youth, remains to be determined.

Some study limitations are noteworthy. This was a secondary cross-sectional analysis in a predominantly African American youth and young adults. Although we acknowledge that the homogeneity of our study participants limits the generalizability of findings, our population demographics reflect the burden of disease in youth at risk for T2DM. Therefore, it was imperative that these biomarkers of interest were evaluated in a population-specific manner that included youth with preDM, and T2DM of African ancestry in whom traditional markers of insulin resistance (*e.g.* TG/HDL ratio) are imprecise. Larger studies will be required to validate NMR biomarker prediction in a multi-ethnic youth population. In addition, nutritional habits were not collected during this study and the influence of diet on glycine, BCAAs, and serum lipid concentrations could not be evaluated. Nevertheless, our study design reflected ‘real-world’ clinical conditions in which detailed dietary profiles are not available. Another limitation of this analysis was the use of frozen samples that may have underestimated biomarker levels (23), though in a consistent manner across all study samples. To minimize bias related to systematic underestimation, all samples were processed after only one freeze–thaw cycle and batched for analysis. Additionally, we acknowledge that HOMA-IR was not a gold-standard measure of insulin resistance, and its use in individuals with dysglycemia is not without controversy (37, 53). We justified choosing HOMA-IR *a priori* because it allowed us to determine associations with a commonly used fasting research index in large group of at-risk youth, in which invasive measurements of a insulin resistance were not possible.

## Conclusion

The ability to risk-stratify youth with insulin resistance, especially in the clinical setting, has been challenging. This study demonstrated the utility of NMR-derived biomarkers obtained from the clinical LipoProfile^®^ lipid testing in a high-risk pediatric population across the spectrum of glucose tolerance. LPIR and GlycA were superior biomarkers of glycemia and insulin resistance compared to BCAA and glycine. LPIR was the strongest correlate of insulin resistance and a value of >44 was associated with the highest cardiometabolic risk—findings that strongly support the versatility of using a universal LPIR scoring system across race/ethnicities in both children and adults. Biomarkers of insulin resistance that can be applied across different racial and ethnic groups in both youth and adults, with a single blood draw, could prove extremely useful in a clinical setting. LPIR, alone or in combination with GlycA, is potentially an important addition to the clinician’s toolbox and should be explored as predictive tools of cardiometabolic risk in prospective studies of diverse youth populations.

## Data Availability Statement

A limited dataset containing the raw data supporting the conclusions of this article will be made available by the authors upon request. 

## Ethics Statement

The studies involving human participants were reviewed and approved by the Institutional Review Board at Baylor College of Medicine, NIH, and CHOP. Written informed consent to participate in this study was provided by the participants’ legal guardian/next of kin.

## Author Contributions

STC conceptualized and designed the study, recruited, and collected the data, conducted the analysis, and wrote the manuscript. STM conceptualized the study, conducted the analysis, and wrote the manuscript. SNM designed the study, recruited and collected the data, and edited and revised the manuscript. AGM, CKC, AV-P, JMD, VRS, MLS, and JDO contributed to study design, and data collection, and revised and edited the manuscript. STC is the guarantor of this work and, as such, had full access to all data in the study and takes responsibility for the integrity of the data and the accuracy of the data analysis. All authors contributed to the article and approved the submitted version.

## Funding 

The Division of Intramural Research supports SC (National Institute of Diabetes & Digestive & Kidney Diseases), MS (the NIH Clinical Center), and AM (National Institute of Child Health and Development). SM received NIH funding (K23 PA05143). The study was presented as a moderated poster at the 80^th^ Scientific Sessions of the American Diabetes Association, June 2020.

## Conflict of Interest

JO was employed by the company Laboratory Corporation of America Holdings (LabCorp), Morrisville, NC, USA.

The remaining authors declare that the research was conducted in the absence of any commercial or financial relationships that could be construed as a potential conflict of interest.
