# Supplementary material for: Nuclear Magnetic Resonance Derived Biomarkers for Evaluating Cardiometabolic Risk in Youth and Young Adults Across the Spectrum of Glucose Tolerance
Source: Front Endocrinol (Lausanne). 2021 May 18;12:665292. doi: 10.3389/fendo.2021.665292 (PMC8167058; doi:10.3389/fendo.2021.665292)
Supplement: Supplementary file 1 [file Table_1.docx]

**Supplemental Materials**

**Supplemental Table 1. Correlation of Biomarkers with Age**

|  | n | r | *P-*value |
| --- | --- | --- | --- |
| LPIR | 186 | 0.0149 | 0.8399 |
| GlycA | 186 | 0.0834 | 0.2577 |
| Glycine | 184 | 0.0154 | 0.8353 |
| BCAA | 186 | 0.1236 | 0.0927 |

**Supplemental Table 2 Correlation of Biomarkers with Entire Cohort vs. African American Participants Only**

|  | **LPIR score** | | | | GlycA (μmol/L) | | | ***Ln*BCAA (μmol/L)** | | | **Glycine**  **(μmol/L)** | |
| --- | --- | --- | --- | --- | --- | --- | --- | --- | --- | --- | --- | --- |
| Log HOMA-IR | | | | | | | | | | | | |
|  | | Entire cohort | AA only | Entire cohort | | AA only | Entire cohort | | AA only | Entire cohort | | AA only |
| **r** | | 0.6231 | 0.6410 | 0.4408 | | 0.4208 | 0.2482 | | 0.2504 | -0.3634 | | -0.4163 |
| ***P*-value** | | **<0.001** | **<0.001** | **<0.001** | | **<0.001** | **0.001** | | **0.0028** | **<0.001** | | **<0.001** |
| **n** | | 184 | 141 | 184 | | 141 | 184 | | 141 | 182 | | 140 |
| Log HOMA2-IR | | | | | | | | | | | | |
|  | | **Entire cohort** | **AA only** | **Entire cohort** | | **AA only** | **Entire cohort** | | **AA only** | **Entire cohort** | | **AA only** |
| **r** | | 0.6006 | 0.6169 | 0.4612 | | 0.4519 | 0.2091 | | 0.1942 | -0.3577 | | -0.4130 |
| ***P*-value** | | **<0.001** | **<0.001** | **<0.001** | | **<0.001** | **0.0044** | | **0.0211** | **<0.001** | | **<0.001** |
| **n** | | 184 | 141 | 184 | | 141 | 184 | | 141 | 182 | | 140 |
| HbA1c | | | | | | | | | | | | |
|  | | Entire cohort | AA only | Entire cohort | | AA only | Entire cohort | | AA only | Entire cohort | | AA only |
| **r** | | 0.4795 | 0.4855 | 0.2811 | | 0.2720 | 0.3186 | | 0.0372 | -0.3168 | | -0.3611 |
| ***P*-value** | | **<0.001** | **<0.001** | **<0.001** | | **0.0012** | **<0.001** | | **<0.001** | **<0.001** | | **<0.001** |
| **n** | | 179 | 139 | 179 | | 139 | 179 | | 139 | 177 | | 138 |

**Supplemental Table 3: Correlations of NMR-derived Biomarkers with Insulin Resistance and Glycemia**

|  | **LPIR score** | GlycA  **(μmol/L)** | ***Ln*BCAA (μmol/L)** | **Glycine**  **(μmol/L)** |
| --- | --- | --- | --- | --- |
| Log HOMA-IR | | | | |
| **r** | 0.6231 | 0.4408 | 0.2482 | -0.3634 |
| ***P*-value** | **<0.001** | **<0.001** | **0.001** | **<0.001** |
| **n** | 184 | 184 | 184 | 182 |
| Log HOMA2-IR | | | | |
| **r** | 0.6006 | 0.4612 | 0.2091 | -0.3577 |
| ***P*-value** | **<0.001** | **<0.001** | **0.0044** | **<0.001** |
| **n** | 184 | 184 | 184 | 182 |
|  |  |  |  |  |
| HbA1c | | | | |
| **r** | 0.4795 | 0.2811 | 0.3186 | -0.3168 |
| ***P*-value** | **<0.001** | **<0.001** | **<0.001** | **<0.001** |
| **n** | 179 | 179 | 179 | 177 |

LPIR: Lipoprotein Insulin Resistance Index; BCAA: Branched-chain amino acid; HOMA-IR: homeostatic model of insulin resistance; HOMA2-IR: revised homeostatic model of insulin resistance; *Ln*: natural logarithm; r: Spearman’s correlation coefficient; n: total number
